# Clinical Patterns and Treatment Outcomes of Selected Benign Anorectal Conditions in a Tertiary Care Hospital in Vijayapura, India: A Retrospective Observational Study

**DOI:** 10.7759/cureus.105512

**Published:** 2026-03-19

**Authors:** Nishikant N Gujar, Mohammed Sohail Malkhed, Krishna Vemuri

**Affiliations:** 1 General Surgery, Al-Ameen Medical College, Vijayapura, IND

**Keywords:** anal fissure, anorectal abscess, clinical profile, fistula-in-ano, perianal sepsis, recurrence, tertiary care, treatment outcome

## Abstract

Background: Anal fissure, fistula-in-ano, and anorectal abscess are common benign anorectal conditions that contribute substantially to pain, morbidity, repeated hospital visits, and procedure-related workload in surgical practice. Because these conditions differ in presentation, anatomical characteristics, and treatment pathways, institution-specific data on their clinical profile and early outcomes are useful for improving decision-making and follow-up in tertiary care settings.

Objectives: To describe the clinical profile and symptom pattern of patients with anal fissure, fistula-in-ano, and anorectal abscess, and to assess treatment outcomes and early recurrence in a tertiary care hospital.

Methods: A hospital-based retrospective observational study was conducted in the Department of General Surgery, Al-Ameen Medical College and its associated teaching hospital, Vijayapura, Karnataka, using hospital records from the study period 2024-2025. Adult patients aged 18 years and above with a documented diagnosis of anal fissure, fistula-in-ano, or anorectal abscess were included. Haemorrhoidal disease was not included because the study was intentionally restricted to these three conditions, which differ from haemorrhoids in pathophysiology, clinical course, management algorithms, and outcome assessment. Demographic details, presenting symptoms, disease subtypes, treatment received, and follow-up outcomes were extracted from case records using a structured data collection format. Outcome measures included symptom resolution, wound healing, postoperative complications, and recurrence. Categorical variables were analysed using the chi-square test, with p < 0.05 considered statistically significant.

Results: A total of 162 patients were included. Anal fissure accounted for 58 cases (35.8%), fistula-in-ano for 54 (33.3%), and anorectal abscess for 50 (30.9%). Chronic fissure was seen in 39/58 (67.2%). Simple low fistulas comprised 37/54 (68.5%), while 17/54 (31.5%) were complex. Perianal abscess was the most frequent abscess subtype (76%). Pain was common in fissure and abscess, discharge predominated in fistula, and swelling with fever was strongly associated with abscess (p < 0.05). Overall, symptom relief exceeded 85% across groups. Recurrence was low after fissure surgery and comparatively higher in fistula cases.

Conclusion: Selected benign anorectal conditions presented in nearly comparable proportions, with distinct symptom profiles that supported clinical diagnosis. Standard treatment protocols achieved high short-term success, while fistula disease showed a greater tendency toward recurrence, underscoring the need for careful anatomical assessment and follow-up.

## Introduction

Anal fissure, fistula-in-ano, and anorectal abscess are common benign anorectal conditions encountered in surgical outpatient and emergency practice. Although they are rarely life-threatening, they carry a disproportionate burden of pain, anxiety, loss of workdays, and repeated hospital visits. In routine practice, these entities often overlap along a clinical continuum-anorectal abscess commonly represents acute sepsis, while fistula-in-ano is frequently the chronic sequela of cryptoglandular infection, and fissure represents a distinct but equally disabling pathology driven by anodermal injury and sphincter hypertonicity [1-5].

The clinical presentation of perianal abscess is usually dramatic, with severe perianal pain, fever, and local swelling, requiring timely drainage to prevent progression and systemic complications. Despite adequate incision and drainage, a considerable proportion of patients later develop fistula-in-ano, which may lead to persistent discharge, recurrent sepsis, and repeat procedures [6-11]. Contemporary evidence continues to debate whether adjuvant antibiotic therapy after drainage meaningfully reduces later fistula formation, and practice patterns remain inconsistent across centres and patient subgroups [11,12]. This uncertainty becomes more relevant in tertiary hospitals where the case mix includes diabetics, immunocompromised patients, recurrent disease, and referrals after prior procedures-groups in whom outcomes may differ from textbook expectations.

Fistula-in-ano remains one of the most technically challenging benign colorectal problems because treatment success must be balanced against preservation of continence. Low, uncomplicated fistulas can often be treated successfully with fistulotomy, whereas more complex tracts usually call for sphincter-preserving procedures, such as ligation of the intersphincteric fistula tract (LIFT), advancement flap repair, video-assisted techniques, or laser closure, all of which differ in their healing outcomes and risk of recurrence [1,3-7]. Recent syntheses highlight that no single technique consistently outperforms others across all complex fistula patterns, and outcomes depend heavily on fistula anatomy, prior sepsis control, internal opening management, and surgeon experience [5-7]. Newer adjuncts, including platelet-rich plasma and laser-based therapies, are increasingly reported, yet real-world effectiveness varies and standardisation is still evolving [6-10].

Although anal fissure is a small lesion confined to the anoderm, its current burden in clinical practice is considerable because the pain is often intense, especially during and after defecation, and is frequently accompanied by bleeding, bowel avoidance, constipation, and significant discomfort in daily life. The condition can disturb appetite, sleep, work routine, and psychological well-being, as many patients develop a fear of passing stools and delay seeking care because of embarrassment or anticipation of pain. Management usually begins with correction of bowel habits, regulation of stool consistency, topical smooth muscle relaxants, and chemical sphincterotomy, such as botulinum toxin, whereas lateral internal sphincterotomy is generally reserved for persistent or chronic fissures that fail to improve with conservative treatment [13-19]. Even in the management of anal fissure, treatment approaches have gradually evolved in recent years. This shift reflects increasing awareness of the risk of continence impairment after surgery, particularly in susceptible patients, along with a growing preference for conservative or minimally invasive options that reduce pain, hospital stay, and recovery time. In addition, the wider availability of day-care procedures has influenced clinical decision-making, making it possible to offer effective treatment with less disruption to the patient’s daily routine. As a result, current practice is moving toward a more individualised approach in which symptom severity, chronicity, risk of sphincter injury, and patient expectations are all taken into account before selecting the most appropriate intervention [13,14,19]. Evidence comparing medical therapy and surgical options continues to expand, but local outcome data are essential because adherence, follow-up, and comorbidity profiles can substantially influence healing and recurrence [15-19].

In India, tertiary care hospitals manage a wide spectrum of perianal disease severity, from first-episode abscesses presenting to emergency services to complex recurrent fistulas referred after multiple failed procedures. Despite this high clinical load, many centres lack consolidated, region-specific audits describing (i) the clinical pattern at presentation, (ii) distribution of disease subtypes and complexity, (iii) commonly used treatment pathways, and (iv) short- and medium-term outcomes, such as symptom resolution, wound healing time, recurrence, fistula formation after abscess drainage, complications, and need for re-intervention. Such evidence is necessary to benchmark outcomes, refine institutional protocols, rationalise antibiotic use, and guide patient counselling based on realistic expectations rather than extrapolated data.

Against this background, the present study evaluates the clinical pattern and treatment outcomes of three selected benign anorectal conditions, namely anal fissure, fistula-in-ano, and anorectal abscess, managed in a tertiary care hospital. Haemorrhoidal disease was not included because it represents a distinct clinical entity with separate grading systems, management pathways, and outcome measures.

## Materials and methods

Study design and setting

The present study was designed as a hospital-based retrospective observational study conducted in the Department of General Surgery, Al-Ameen Medical College and its attached teaching hospital, Vijayapura, Karnataka. A retrospective design was considered appropriate because it allowed evaluation of routinely documented clinical information without interfering with standard patient care and was feasible within the ethical and institutional resource framework of our centre. The analysis was based on hospital records of patients managed during the study period from January 2024 to December 2025. As a tertiary care centre, the hospital serves a broad population from urban and rural regions of North Karnataka and neighbouring districts. The department receives patients through outpatient services, emergency admissions, and referrals from peripheral hospitals, thereby reflecting a wide clinical spectrum ranging from uncomplicated primary anorectal conditions to recurrent and complex cases requiring specialised surgical management.

Study population

The study population comprised adult patients aged 18 years and above with a documented diagnosis of anal fissure, fistula-in-ano, or anorectal abscess based on clinical evaluation and available supporting investigations recorded in the case files. The study was intentionally restricted to these three conditions in order to maintain a more homogeneous analytical framework for comparing clinical presentation, treatment patterns, and early outcomes.

Haemorrhoidal disease was not included because it differs from these conditions in pathophysiology, classification, management pathways, and outcome assessment, and its inclusion would have introduced substantial clinical heterogeneity. Patients with perianal manifestations secondary to Crohn’s disease, tuberculosis, anorectal malignancy, or prior radiation exposure were excluded to preserve uniformity of disease mechanisms. Pregnant women and records with incomplete documentation of key study variables were also excluded. Eligible cases were identified retrospectively from hospital records of patients treated during the study period.

Ethical considerations

The protocol for this retrospective record-based study was reviewed and approved by the Institutional Ethics Committee of Al-Ameen Medical College, Vijayapura. The ethics approval pertains to the retrospective study protocol and does not correspond to the period during which the patient's received treatment. Data were extracted from existing hospital records of patients managed during 2024-2025, and no change in patient care was made for research purposes. All data were anonymised before analysis, and confidentiality was maintained throughout data handling, compilation, and interpretation.

Data collection procedure

Data were collected retrospectively from hospital case records using a structured and pretested data extraction form to ensure uniformity and completeness of recording. Information on demographic details, presenting complaints, symptom duration, and relevant clinical history was retrieved from available records. Symptoms such as perianal pain, swelling, discharge, bleeding per rectum, fever, and altered bowel habits were noted wherever documented. Particular attention was given to associated factors, including constipation, straining during defecation, previous similar episodes, and prior surgical intervention for anorectal disease, in order to identify recurrent cases.

Details of general clinical assessment documented in the case sheets were also reviewed, including comorbid conditions such as diabetes mellitus and hypertension, which may influence infection risk, wound healing, and postoperative recovery. Findings of local examination of the perianal region, including swelling, erythema, discharge, external openings, sentinel pile, and induration, were extracted from the records. Documentation of digital rectal examination, including sphincter tone, tenderness, induration, and suspected internal opening when available, was also noted. Proctoscopic findings were recorded in cases in which the procedure had been performed and documented in the hospital records.

In patients diagnosed with fistula-in-ano, details on the number and position of external openings were obtained from the clinical notes, including descriptions using the clock-face method whenever available. Information on the presumed internal opening and direction of the tract was also collected from the recorded examination findings or operative notes. Imaging investigations, such as ultrasonography or magnetic resonance imaging, were noted in selected cases, particularly when the records indicated complex fistula, recurrent disease, or multiple tracts, as these investigations were performed to define the anatomical extent before intervention.

Diagnostic classification

Based on the clinical findings documented in the medical records and the available supporting investigations, patients were categorised into three primary diagnostic groups: anal fissure, fistula-in-ano, and anorectal abscess. Anal fissures were further classified as acute or chronic. Chronic fissure was identified from documented features such as a sentinel pile, hypertrophied anal papilla, or visible internal sphincter fibres.

Fistula-in-ano cases were categorised as simple or complex according to the information recorded regarding sphincter involvement, number of tracts, and recurrence status. Simple fistulas were generally low fistulas involving minimal sphincter muscle, whereas complex fistulas included high tracts, multiple branches, anterior fistulas in females, or recurrent disease.

Anorectal abscesses were classified according to anatomical location, including perianal, ischiorectal, intersphincteric, and supralevator types, based on clinical documentation and radiological findings where available.

Treatment protocol

This study did not impose any study-specific intervention. Management had been provided as part of routine surgical care according to standard departmental practice and was subsequently reviewed from the medical records.

Patients with acute anal fissure had generally been managed conservatively with dietary advice aimed at increasing fibre intake, adequate hydration, stool softeners, warm sitz baths, and topical agents such as nitrate or calcium channel blocker preparations to reduce internal sphincter spasm. Patients with chronic fissure who did not respond adequately to medical therapy had undergone surgical intervention in the form of lateral internal sphincterotomy under appropriate anaesthesia, as documented in the records.

All patients diagnosed with anorectal abscess had undergone incision and drainage under suitable anaesthesia as part of standard treatment. The procedure generally involved evacuation of pus and breakdown of loculi to ensure adequate drainage. In selected patients, particularly those with diabetes, systemic signs of infection, or extensive cellulitis, antibiotic therapy had also been administered in addition to surgical drainage. Pus culture and sensitivity testing had been performed where clinically indicated and documented.

In fistula-in-ano, the choice of procedure had been guided by fistula anatomy and the extent of sphincter involvement. Simple low fistulas had been treated with fistulotomy or fistulectomy, whereas in complex fistulas, sphincter-preserving procedures, such as seton placement, had been considered to reduce the risk of continence disturbance. The operative technique and intraoperative findings were obtained from the operative records.

Postoperative care and follow-up

Postoperative care had been provided according to the routine protocols of the department. This generally included analgesics, antibiotics where indicated, advice regarding local hygiene and sitz baths, and dietary guidance to maintain soft stools and reduce straining. Wound care instructions were given as part of usual discharge counselling, and dressings were applied either at the hospital or at peripheral centres, depending on patient convenience and accessibility.

Follow-up information was obtained retrospectively from outpatient records. As per usual departmental practice, patients were typically reviewed in the surgical outpatient department at weekly intervals during the first month and subsequently at monthly intervals for at least three months, wherever such visits were available in the records. During follow-up, wound-healing status, persistence or resolution of discharge, pain, postoperative complications, and recurrence of symptoms were noted from the documented clinical entries.

Outcome measures

The primary outcomes assessed were resolution of symptoms, time required for wound healing where documented, recurrence of abscess or fistula, and development of fistula following abscess drainage. In patients who underwent surgery for fistula-in-ano, healing of the tract and absence of persistent discharge during the available follow-up period were taken as indicators of successful treatment.

Secondary outcomes included postoperative complications such as bleeding, wound infection, delayed healing, and symptoms suggestive of anal incontinence when documented in the records. Continence status was evaluated on the basis of patient-reported complaints recorded during follow-up visits rather than by formal scoring tools.

Statistical analysis

All relevant data were extracted from hospital records, anonymised, coded, and entered into Microsoft Excel (Microsoft Corporation, Redmond, WA, USA). Statistical analysis was performed using IBM SPSS Statistics (IBM Corp., Armonk, NY, USA). Data cleaning was undertaken before analysis to identify duplicate entries, out-of-range values, and internal inconsistencies across variables.

Descriptive statistics were used to summarise the study cohort and the distribution of diagnoses. Categorical variables such as sex, symptom profile, disease subtypes, management approach, complications, and recurrence were presented as frequencies and percentages. Continuous variables, such as age, were summarised using mean and standard deviation when normally distributed, or median with interquartile range when the distribution was skewed.

For inferential analysis, cross-tabulations were prepared to compare clinical features and outcomes across the diagnostic groups. The chi-square test was used to assess associations between categorical variables, while Fisher’s exact test was applied when expected cell counts were small. All tests were two-tailed, and a p-value of less than 0.05 was considered statistically significant. Findings were interpreted in a clinical context in addition to statistical significance.

## Results

Demographic profile

Overall, the majority of patients clustered in the 31-50-year age group, accounting for just over half of the total sample, while the ≤30 and >50-year groups formed smaller but comparable proportions. When the age distribution was compared across fissure, fistula, and abscess groups, the difference was not statistically meaningful (χ² = 3.12, p = 0.79), indicating that the three conditions occurred across similar adult age ranges in this cohort (Table 1). In contrast, gender showed a clear pattern. Males constituted nearly three-fourths of the entire study population and were particularly predominant in fistula and abscess cases. The association between gender and disease category reached statistical significance (χ² = 8.94, p = 0.011), suggesting that fistula-in-ano and anorectal abscess were more frequently observed among male patients in this tertiary care setting, while fissure showed a relatively higher female representation compared with the other two groups.

**Table 1:** Age and gender distribution of study participants. Values are presented as n (%). P values were calculated using the Pearson chi-square test. p < 0.05 was considered statistically significant (*).

Variable	Fissure (n = 58)	Fistula (n = 54)	Abscess (n = 50)	Total (n = 162)	χ² value	p-value
Age ≤30 years	14 (24.1%)	11 (20.4%)	9 (18.0%)	34 (21.0%)	3.12	0.79
31–50 years	31 (53.4%)	30 (55.6%)	28 (56.0%)	89 (54.9%)	-	-
>50 years	13 (22.4%)	13 (24.0%)	13 (26.0%)	39 (24.1%)	-	-
Male	36 (62.1%)	44 (81.5%)	40 (80.0%)	120 (74.1%)	8.94	0.011*
Female	22 (37.9%)	10 (18.5%)	10 (20.0%)	42 (25.9%)		

Clinical presentation

Pain was the most consistent complaint, and it was nearly universal among fissure and abscess patients, whereas a relatively lower, but still substantial, proportion of fistula patients reported pain; this difference was statistically significant (χ² = 14.72, p = 0.001). Discharge emerged as the most discriminatory symptom: it was strongly concentrated in fistula-in-ano, where almost all patients had discharge, while it was uncommon in fissure and present in a smaller fraction of abscess cases. This association was highly significant (χ² = 96.84, p < 0.001), supporting discharge as a key pointer toward fistula in routine clinical evaluation. Swelling followed a similar diagnostic gradient, being overwhelmingly present in abscess cases, less frequent in fistula cases, and rare in fissure cases; this pattern was again highly significant (χ² = 104.33, p < 0.001). Fever also showed a strong association with abscess, with more than half of patients reporting fever, compared with much smaller proportions in the fistula and fissure groups, and the difference was statistically significant (χ² = 63.91, p < 0.001) (Table 2). Taken together, the table reflects a clinically intuitive but clearly quantified picture -- fissure and abscess are largely pain-dominant presentations, fistula is discharge-dominant, and abscess is characterised by swelling and systemic symptoms more often than the other two conditions.

**Table 2:** Presenting symptoms according to diagnosis. Values are presented as n (%). P values were obtained using the Pearson chi-square test for comparison across the three diagnostic groups. p < 0.05 was considered statistically significant (*).

Symptom	Fissure (n = 58)	Fistula (n = 54)	Abscess (n = 50)	χ² value	p-value
Pain	55 (94.8%)	41 (75.9%)	48 (96.0%)	14.72	0.001*
Discharge	8 (13.8%)	50 (92.6%)	15 (30.0%)	96.84	<0.001*
Swelling	5 (8.6%)	18 (33.3%)	47 (94.0%)	104.33	<0.001*
Fever	2 (3.4%)	9 (16.7%)	29 (58.0%)	63.91	<0.001*

Type of disease distribution

**Figure 1:**
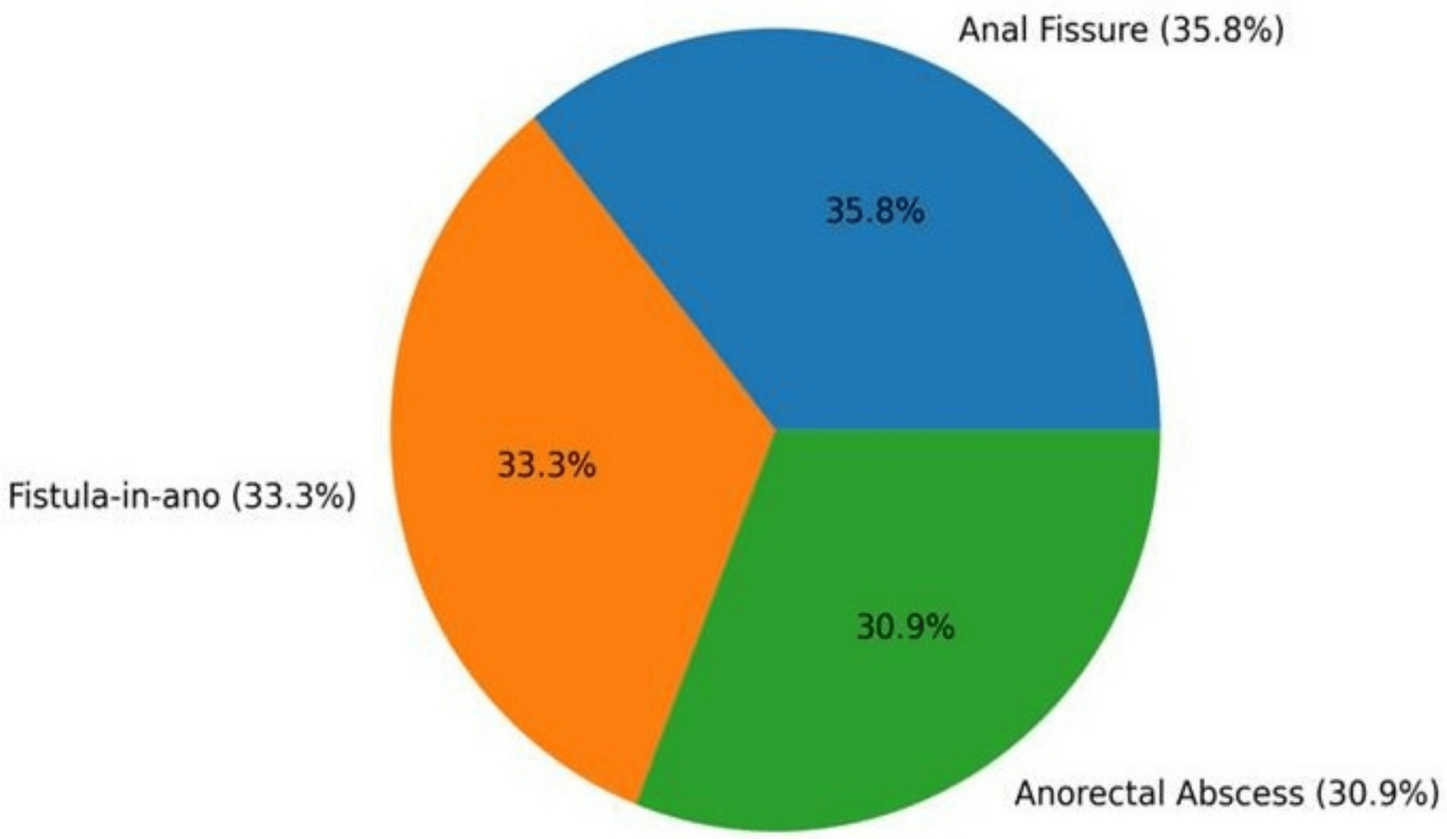
Distribution of perianal diseases among study participants.

In the present series of 162 patients with perianal diseases, anal fissure was the most frequently encountered condition, accounting for 58 cases (35.8%), followed closely by fistula-in-ano with 54 cases (33.3%) and anorectal abscess with 50 cases (30.9%). A notable proportion of fissure patients had established disease at presentation, as 39 cases (67.2%) were classified as chronic. Among the fistula group, the majority were simple low fistulas (37 cases; 68.5%), while complex fistulas constituted 17 cases (31.5%), indicating that nearly one-third required more careful operative planning to protect sphincter function (Figure 1). With respect to anorectal abscess, perianal abscess was the predominant subtype, forming 76% of abscess presentations, highlighting that superficial perianal sepsis was the most common acute pattern in this cohort.

Symptom pattern across disease types

**Figure 2:**
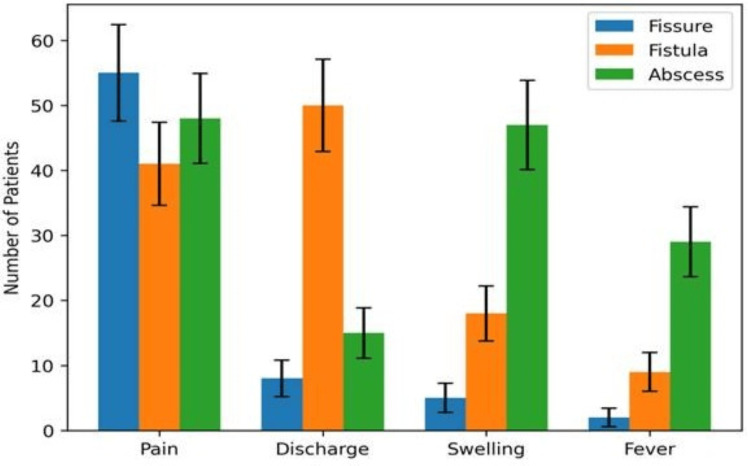
Symptom pattern across disease types.

Figure 2 highlights a distinct symptom profile for each perianal condition in this cohort. Pain was the most consistent feature overall and was reported by almost all patients with anal fissure and anorectal abscess, while a comparatively smaller proportion of fistula cases complained of pain. Discharge showed a striking association with fistula-in-ano, where it was seen in the vast majority of patients, but it was infrequent in fissure and present only in a smaller subset of abscess cases. Swelling was the hallmark of an abscess, occurring in nearly all abscess presentations, whereas it appeared less often in a fistula and was rarely noted in a fissure. Fever followed a similar trend, being reported mainly by abscess patients and only occasionally in the other two groups. The SEM error bars included in the clustered bars give a sense of dispersion around the observed counts and reinforce the overall pattern -- discharge predominates in fistula, swelling and fever are typical of abscess, and pain is a near-universal complaint in fissure and abscess.

Treatment modality and immediate outcome

Nearly two-fifths of fissure cases were managed conservatively, reflecting the role of non-operative measures in early or less severe fissures. However, a larger share required surgery, which is consistent with chronic fissure presentations where definitive sphincter-relaxing procedures are commonly needed for sustained relief. The immediate response pattern in fissure patients showed a statistically significant association between treatment approach and symptomatic improvement (χ² = 6.72, p = 0.035), indicating that the chosen management strategy had a measurable relationship with early relief in this group (Table 3). In contrast, all fistula and abscess patients underwent surgical treatment, reflecting the standard of care-definitive tract management for fistula and incision and drainage for abscess. Across the three groups, the proportion achieving complete early symptom relief remained high, exceeding 85% in each category, which suggests that the department’s routine management pathways were effective in achieving short-term clinical improvement in most patients.

**Table 3:** Treatment modalities and immediate outcome. Values are presented as n (%). P values were calculated using the Pearson chi-square test. p < 0.05 was considered statistically significant (*).

Diagnosis	Conservative	Surgical	Complete relief (%)	χ² value	p-value
Fissure (n = 58)	24 (41.4%)	34 (58.6%)	52 (89.7%)	6.72	0.035*
Fistula (n = 54)	0	54 (100%)	46 (85.2%)		
Abscess (n = 50)	0	50 (100%)	45 (90.0%)		

Postoperative complications and recurrence 

Delayed wound healing was observed most often in fistula cases, followed by abscess and then fissure surgery, but the difference across groups did not reach statistical significance (χ² = 1.21, p = 0.547). A similar pattern was noted for postoperative infection, which appeared somewhat more frequent after fistula surgery compared to fissure and abscess procedures, though this variation was also not statistically significant (χ² = 0.77, p = 0.682). Recurrence was numerically highest among fistula patients, with smaller proportions in abscess and very low recurrence after fissure surgery; however, the overall association between diagnosis and recurrence did not meet the conventional threshold for significance in this sample (χ² = 3.23, p = 0.199) (Table 4). In practical terms, the table indicates that while fistula cases tended to show relatively higher rates of delayed healing and recurrence, the study size and event counts were not sufficient to confirm these differences statistically.

**Table 4:** Postoperative complications and recurrence. Values are presented as n (%). Group-wise differences were assessed using the Pearson chi-square test. A two-tailed p-value <0.05 was considered statistically significant.

Outcome	Fissure (n = 34 surgical)	Fistula (n = 54)	Abscess (n = 50)	χ² value	p-value
Delayed healing	3 (8.8%)	9 (16.7%)	6 (12.0%)	1.21	0.547
Recurrence	1 (2.9%)	8 (14.8%)	5 (10.0%)	3.23	0.199
Post-op infection	2 (5.8%)	6 (11.1%)	4 (8.0%)	0.77	0.682

## Discussion

The present study evaluated the clinical presentation and short-term treatment outcomes of patients managed for anal fissure, fistula-in-ano, and anorectal abscess in a tertiary care surgical unit. Among the three conditions, anal fissure constituted the largest diagnostic group, followed closely by fistula-in-ano and anorectal abscess. The relatively comparable distribution of these disorders suggests that benign anorectal conditions represent a regular and substantial component of everyday surgical practice. This observation is consistent with current guidance and institutional reports that regard these conditions as a major part of routine proctologic workload in general surgery settings [1,2].

A marked male predominance was observed in the overall cohort, particularly among patients with fistula-in-ano and anorectal abscess, whereas the sex distribution in fissure was relatively less pronounced. This pattern is in broad agreement with previous reports, where cryptoglandular sepsis and its sequelae have been documented more often in men, while anal fissure has shown a more balanced sex distribution in some populations [1,20]. In contrast, age distribution across the three diagnostic groups did not differ substantially. Most patients were in the economically productive age group, indicating that these disorders affect not only health and quality of life but also day-to-day functioning and work productivity. Similar age patterns have been reported in hospital-based studies of perianal sepsis and fistulous disease [1,2].

The symptom profile in the present study showed clear clinical separation across the diagnostic categories. Pain was the dominant symptom in fissure and abscess, discharge was strongly associated with fistula-in-ano, and swelling and fever were largely concentrated in abscess cases. These findings are biologically plausible and reflect the underlying disease process. Pain in fissure is typically related to anodermal injury and internal sphincter spasm, anorectal abscess presents as an acute suppurative condition, and fistula-in-ano more commonly manifests with persistent or intermittent tract-related discharge rather than systemic inflammatory features [14,20]. The statistically significant variation in symptom distribution across the three groups reinforces the importance of careful history-taking and local examination in the initial diagnosis of benign anorectal disease.

A notable proportion of fissure patients in our series had chronic disease at presentation. This is clinically important because chronic fissure is less likely to respond completely to simple conservative measures and often requires escalation of therapy. Current evidence supports a stepwise approach that begins with bowel regulation, dietary modification, sitz baths, and topical sphincter-relaxing agents, while surgical treatment is usually reserved for persistent or chronic cases [15-19]. In the present study, fissure patients demonstrated high rates of symptom relief after definitive treatment and only low recurrence. These findings are in line with published evidence showing that lateral internal sphincterotomy provides dependable healing in appropriately selected patients with chronic fissure, whereas medical management remains a valuable option in early disease and in those who wish to avoid surgery [17-19].

Within the fistula group, most patients had simple low fistulas, while a smaller but important proportion had complex disease. This distribution corresponds with accepted classification-based reports in which low intersphincteric and low transsphincteric fistulas account for the majority of cases, with complex fistulas forming a clinically significant minority [1,3]. Although recurrence was numerically higher in fistula-in-ano than in fissure or abscess, the difference did not reach statistical significance in this sample. Nevertheless, the trend remains clinically meaningful. Outcomes in fistula disease are influenced by several factors, including tract anatomy, degree of sphincter involvement, identification and control of the internal opening, previous surgical intervention, and the presence of active sepsis [3-7]. Recent literature continues to emphasise that no single operative technique is ideal for all cases. Instead, procedures such as fistulotomy, seton placement, LIFT, advancement flap, and minimally invasive methods must be chosen according to fistula characteristics and the need to preserve continence [5-7]. The variability in healing and recurrence reported with laser-based methods and biologic adjuncts also supports the value of centre-specific audits when evaluating treatment performance [6-9].

Management of anorectal abscess in our cohort was primarily surgical drainage, and most patients showed satisfactory early improvement. The predominance of perianal abscess as the most common subtype is consistent with the anatomical distribution described in standard guidelines and surgical literature [2]. However, despite favourable early outcomes, a proportion of patients subsequently developed recurrence or fistula formation. This remains a recognised clinical issue after abscess drainage. The role of postoperative antibiotics in preventing fistula formation continues to be debated, and current evidence generally supports selective rather than routine use. Most recommendations favour antibiotics in patients with diabetes, extensive cellulitis, immunocompromise, systemic infection, or other high-risk features [11,12]. Our findings support the view that prompt and adequate drainage, combined with appropriate follow-up, remains the cornerstone of successful abscess management, while adjuvant antibiotic therapy should be individualised according to clinical context [1,2,11].

Postoperative complications, such as infection and delayed wound healing, were relatively infrequent across the study groups, although delayed healing appeared more common after fistula surgery. This is not unexpected, as fistula procedures often involve larger wounds, chronic inflammation, and, in some cases, more complex tract anatomy. Contemporary studies have similarly identified fistula complexity and patient-related comorbidities as contributors to prolonged healing [10]. Although the present study was not designed to explore predictors of outcome in a multivariable manner, the direction of our findings is consistent with the existing literature. Future studies from the same setting would benefit from stratifying recurrence and healing according to fistula complexity, comorbidity profile, and type of operative technique, especially when comparing sphincter-dividing and sphincter-preserving procedures [4-7,10].

Overall, the findings of this study indicate that routine management protocols in a tertiary care setting can produce favourable short-term outcomes across all three conditions. Early symptom resolution was achieved in the majority of patients, whereas recurrence was lowest in fissure and relatively higher in fistula-in-ano. This general pattern mirrors the broader evidence base and highlights the continued importance of anatomy-based decision-making, appropriate treatment selection, and structured follow-up, particularly in patients with fistulous disease [1-7].

In summary, the present study provides institution-level evidence on the clinical presentation and treatment response of anal fissure, fistula-in-ano, and anorectal abscess in a tertiary care hospital in Karnataka. The symptom patterns observed closely reflect the established behaviour of these conditions described in the literature [14,20]. Anal fissure was predominantly pain-driven and showed excellent outcomes after appropriate treatment escalation [15-19]. Anorectal abscesses commonly present as acute perianal sepsis and respond well to drainage, although recurrence and subsequent fistula formation remain important concerns that warrant follow-up [1,2,11,12]. Fistula-in-ano emerged as the subgroup with greater anatomical complexity and a relatively higher tendency toward recurrence, underscoring the need for careful operative planning and continued postoperative surveillance [3-7,10].

Limitations

Certain limitations should be considered while interpreting the findings of this study. First, since this was a retrospective record-based analysis, the quality of the study depended largely on the completeness and accuracy of documentation in the hospital records. Important variables, such as symptom severity, duration of illness, previous treatment history, associated comorbidities, and detailed operative or postoperative findings, may not have been documented uniformly in all cases. This may have introduced information bias and restricted the depth of analysis.

Second, the study was conducted in a single tertiary care centre, and the patient profile may have been shaped by local referral patterns, institutional practices, and the nature of cases reaching a higher-level surgical unit. As a result, the findings may not fully represent the broader spectrum of benign anorectal disease seen in primary care settings or in other geographic regions.

Third, treatment was delivered as part of routine clinical care rather than according to a standardised research protocol. Therefore, variations in surgeon preference, disease severity, and anatomical complexity, particularly in fistula-in-ano, may have influenced treatment selection as well as short-term outcomes. This limits direct comparison between management approaches.

Follow-up was also relatively short, and some patients may not have returned regularly for postoperative assessment. Consequently, delayed complications, late recurrence, and long-term functional outcomes, especially those related to continence, may have been under-recorded.

Finally, the statistical analysis was largely based on unadjusted comparisons between groups. Therefore, the possibility of residual confounding cannot be excluded, and the observed associations should be interpreted with caution rather than as definitive causal relationships.

## Conclusions

This study describes the clinical profile and short-term outcomes of common perianal conditions managed in a tertiary care setting. Anal fissure was the most frequent diagnosis, with fistula-in-ano and anorectal abscess occurring at comparable rates, together forming a substantial share of the routine surgical workload. Clear symptom patterns were evident: pain was prominent in fissure and abscess, discharge was strongly associated with fistula, and swelling with fever was seen mainly in abscess, underscoring the value of focused history and examination for early diagnosis and timely triage.

Overall outcomes were favourable with standard treatment pathways. A subset of fissure cases improved with conservative care, while chronic fissures often required surgery with good relief and low recurrence. Abscess management with prompt incision and drainage was largely effective, although a small proportion experienced recurrence or subsequent fistula, supporting the need for follow-up after acute sepsis. Fistula cases showed a higher tendency for delayed healing and recurrence, particularly in complex disease, highlighting the importance of anatomy-guided procedures and structured postoperative surveillance.

## References

[1] Reza L, Gottgens K, Kleijnen J (2024). European Society of Coloproctology: guidelines for diagnosis and treatment of cryptoglandular anal fistula. Colorectal Dis.

[2] Amato A, Bottini C, De Nardi P, Giamundo P, Lauretta A, Realis Luc A, Piloni V (2020). Evaluation and management of perianal abscess and anal fistula: SICCR position statement. Tech Coloproctol.

[3] Garg P, Sodhi SS, Garg N (2020). Management of complex cryptoglandular anal fistula: challenges and solutions. Clin Exp Gastroenterol.

[4] Garg P, Bhattacharya K, Yagnik VD, Mahak G (2024). Recent advances in the diagnosis and treatment of complex anal fistula. Ann Coloproctol.

[5] Sierra Fernández I, Balciscueta Coltell Z, Uribe Quintana N (2025). Systematic review and network meta-analysis of cryptoglandular complex anal fistula treatment: evaluation of surgical strategies. Updates Surg.

[6] Wang Y, Rao Q, Ma Y, Li X (2023). Platelet-rich plasma in the treatment of anal fistula: a systematic review and meta-analysis. Int J Colorectal Dis.

[7] Duda JR, de Oliveira LG, Ferreira LF (2025). Effectiveness of laser-based fistula therapies with and without adjunctive measures in anal fistulas management: a systematic review and single-arm meta-analysis. Int J Colorectal Dis.

[8] Tümer H, Bulbuloglu GC (2023). A comparison of laser and fistulotomy techniques in the treatment of fistula-in-ano. Cureus.

[9] Mei Z, Zhang Z, Han Y, Du P, Yang W, Wang Q, Zheng D (2023). Surgical laser therapy for cryptoglandular anal fistula: protocol of a systematic review and meta-analysis. PLoS One.

[10] Mei Z, Du P, Han Y (2025). Risk factors for delayed wound healing after anal fistula surgery: protocol of a meta-analytic study. PLoS One.

[11] Blondin S, Lobo D, Egal A (2025). Antibiotic use during the first episode of acute perianal sepsis: a still-open question. Ann Coloproctol.

[12] van Oostendorp JY, Dekker L, van Dieren S, Bemelman WA, Han-Geurts IJ (2022). Antibiotic Treatment foLlowing surgical drAinage of perianal abScess (ATLAS): protocol for a multicentre, double-blind, placebo-controlled, randomised trial. BMJ Open.

[13] Picciariello A, Tutino R, Gallo G (2024). Temporal trends and treatment patterns in anal fissure management: insights from a multicenter study in Italy. Tech Coloproctol.

[14] Akinmoladun O, Oh W (2024). Management of hemorrhoids and anal fissures. Surg Clin North Am.

[15] Boland PA, Kelly ME, Donlon NE, Bolger JC, Larkin JO, Mehigan BJ, McCormick PH (2020). Management options for chronic anal fissure: a systematic review of randomised controlled trials. Int J Colorectal Dis.

[16] Sierra-Arango F, de la Hoz-Valle J, Espinosa JP, Moreno-Montoya J, Vásquez Roldan M, Pérez-Riveros ED (2023). Clinical outcomes of medical management options for chronic anal fissures in a long-term follow-up: systematic review and meta-analysis. Dig Dis.

[17] Jin JZ, Hardy MO, Unasa H (2022). A systematic review and meta-analysis of the efficacy of topical sphincterotomy treatments for anal fissure. Int J Colorectal Dis.

[18] Asefa Z, Awedew AF (2023). Comparing closed versus open lateral internal sphincterotomy for management of chronic anal fissure: systematic review and meta-analysis of randomised control trials. Sci Rep.

[19] Bonyad A, Zadeh RH, Asgari S (2024). Botulinum toxin injection versus lateral internal sphincterotomy for chronic anal fissure: a meta-analysis of randomized control trials. Langenbecks Arch Surg.

[20] Włodarczyk M, Włodarczyk J, Sobolewska-Włodarczyk A, Trzciński R, Dziki Ł, Fichna J (2021). Current concepts in the pathogenesis of cryptoglandular perianal fistula. J Int Med Res.

